# Sulfasalazine-induced drug reaction with eosinophilia and systemic symptoms (DRESS) coinfected with COVID-19 complicated by hemophagocytic lymphohistiocytosis: a case report

**DOI:** 10.3389/fimmu.2024.1371490

**Published:** 2024-04-15

**Authors:** Mengmeng Li, Furong li, Yang Dai, Yunou Zhang Zeng, Xiaomei Chen

**Affiliations:** ^1^ Department of Dermatology & Venerology, West China Hospital, Sichuan University, Chengdu, China; ^2^ Department of Hematology, West China Hospital, Sichuan University, Chengdu, China

**Keywords:** drug reaction with eosinophilia and systemic symptoms, COVID-19, hemophagocytic lymphohistiocytosis, ebv, CMV

## Abstract

Drug Reaction with Eosinophilia and Systemic Symptoms (DRESS) is characterized by a widespread maculopapular rash, lymphadenopathy, fever, and multisystem involvement. Conversely, hemophagocytic lymphohistiocytosis (HLH) is an infrequent yet critical condition presenting with fever, hepatosplenomegaly, cytopenias, coagulation abnormalities, and elevated inflammatory markers. The overlapping clinical and laboratory features between DRESS and HLH poses a significant diagnostic challenge. Secondary HLH (sHLH) typically occurs in adults triggered by viral infections, malignancies, rheumatologic diseases, or immune deficiencies. Recently, COVID-19 has also been identified as one of the triggers for sHLH. Herein, we present a case of Sulfasalazine-induced DRESS coinfected with COVID-19 that subsequently progressed into HLH. Our patient exhibited common hepatorenal and splenic involvement along with rare cholecystitis and appendicitis. However, a significant improvement was observed upon the addition of etoposide and azathioprine. We hypothesize that excessive activation of the immune system and cytokine storm due to DRESS combined with COVID-19 infection led to more extensive systemic damage resulting in HLH development. This highlights the potential for severe consequences when DRESS coincides with HLH during a COVID-19 infection.

## Introduction

Drug Reaction with Eosinophilia and Systemic Symptoms (DRESS) is a severe condition that typically manifests 1-6 weeks after exposure to certain medications or their metabolites. It is characterized by extensive maculopapular eruptions, lymphadenopathy, fever, and involvement of multiple organs (1). The mortality rate of DRESS ranges from 1.7% to 8.8%, with recurrence in affected individuals increasing the mortality rate to as much as 29% (2, 3). Hemophagocytic lymphohistiocytosis (HLH) is an uncommon yet fatal syndrome marked by fever, hepatosplenomegaly, cytopenias, coagulopathy, and elevated inflammatory markers among other symptoms (4). In adults, studies have estimated the overall mortality rates to be approximately 40-50%, with early mortality being around 20% despite treatment (5, 6).

The pathogenesis of DRESS and HLH remains incompletely understood. DRESS is characterized by a drug-induced Type IV hypersensitivity reaction, involving the activation of drug-specific CD4 and CD8 T cells as well as dysfunction of regulatory T cells. Immune dysregulations in DRESS are manifested by elevated levels of certain human leukocyte antigens (HLA), reactivation of herpesviruses (HHV-6, Epstein-Barr virus (EBV), cytomegalovirus (CMV)), and autoimmune sequelae. HLH, also known as macrophage activation syndrome (MAS) in the context of rheumatologic disease, is a hyperinflammatory condition resulting from excessive phagocytic activity of macrophages on hematopoietic cells. It can be classified into primary or secondary HLH based on its etiology: primary HLH predominantly affects infants and children due to genotypic defects, while sHLH typically occurs in adults triggered by viral infections, malignancies, rheumatologic diseases, or immune deficiencies. Recently, COVID-19 has also been identified as one of the triggers for sHLH (7).

While there is some overlap in pathogenesis, clinical features, and laboratory abnormalities between DRESS and HLH associated with fever, lymphadenopathy, multisystem organ failure, viral infection/reactivation, and abnormal T cell activation, the coexistence of DRESS and HLH is a rare and potentially fatal condition. The most frequently reported drugs implicated in DRESS complicated by HLH include allopurinol, β-lactam antibiotics, and phenobarbital (8). In this case report, we present a patient who developed Sulfasalazine-induced DRESS coinfected with COVID-19 that subsequently evolved into HLH. This case highlights the intricate interplay between drug reactions, viral infection, and immunological responses.

## Case report

**Figure 1:**
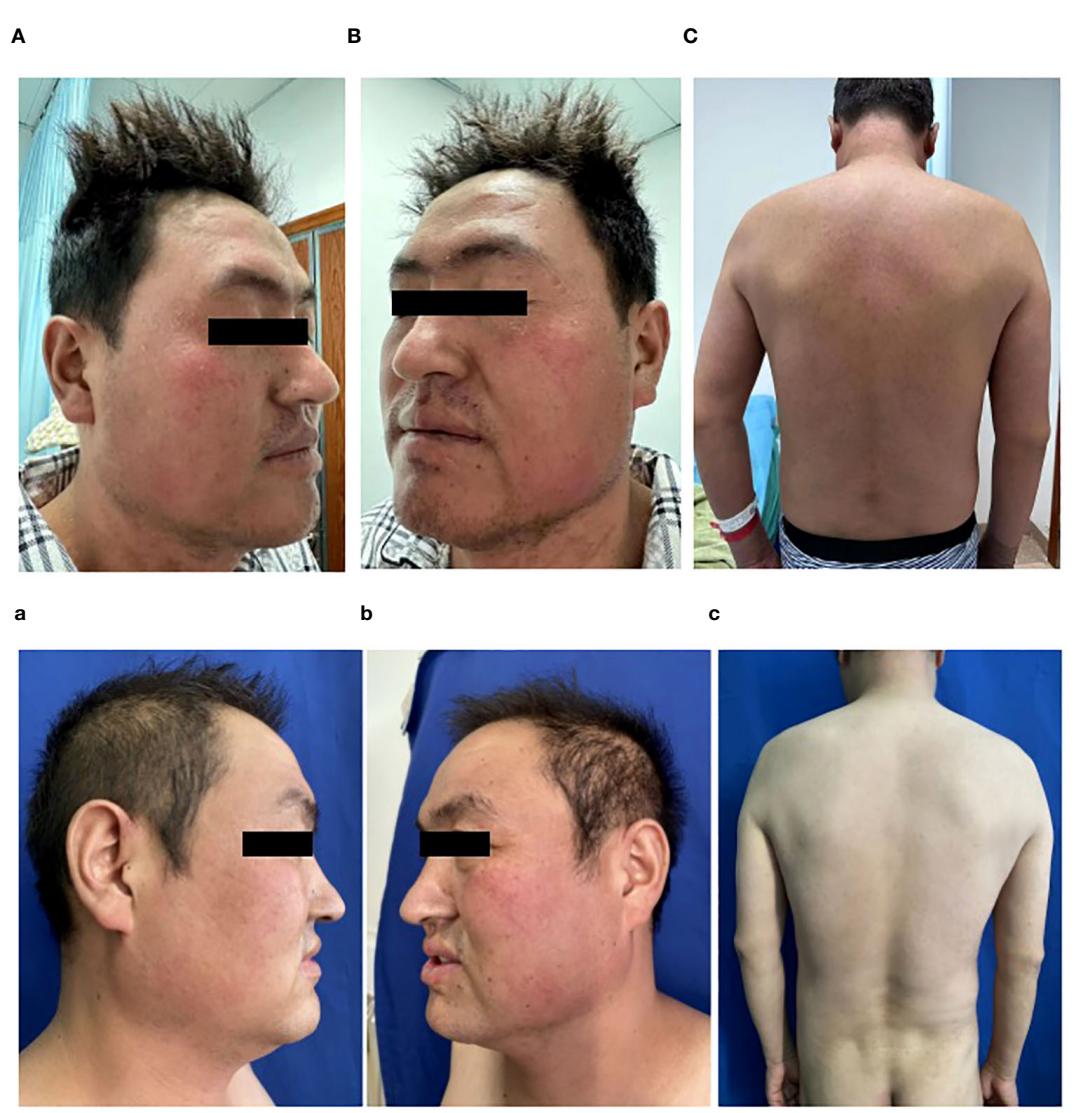
**(A–C)** Diffuse erythema and scale over face and the trunk, on admission; **(a–c)** Post-treatment improvement showing only scattered erythema on the face and almost complete improvement over the trunk.

**Figure 2:**
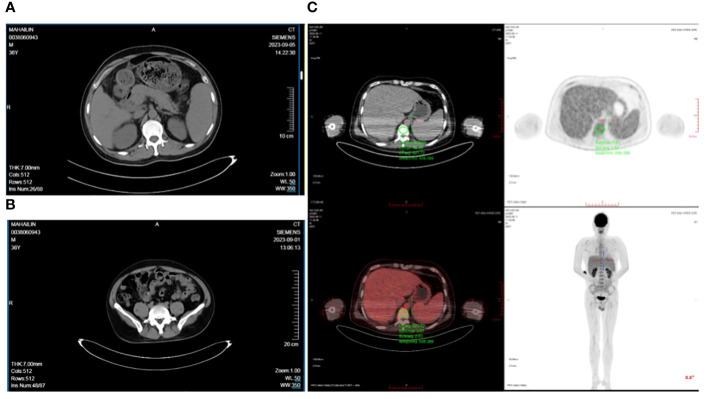
**(A)** The gallbladder wall was edematous, thickened, and rough with a slightly blurred surrounding fat space suggesting acute cholecystitis, **(B)** The appendix was slightly thickened, about 1.2 cm at its thickest, with a blurred surrounding fat space and multiple striated shadows, **(C)** PET-CT scans did not reveal any definitive signs of malignancy.

**Figure 3:**
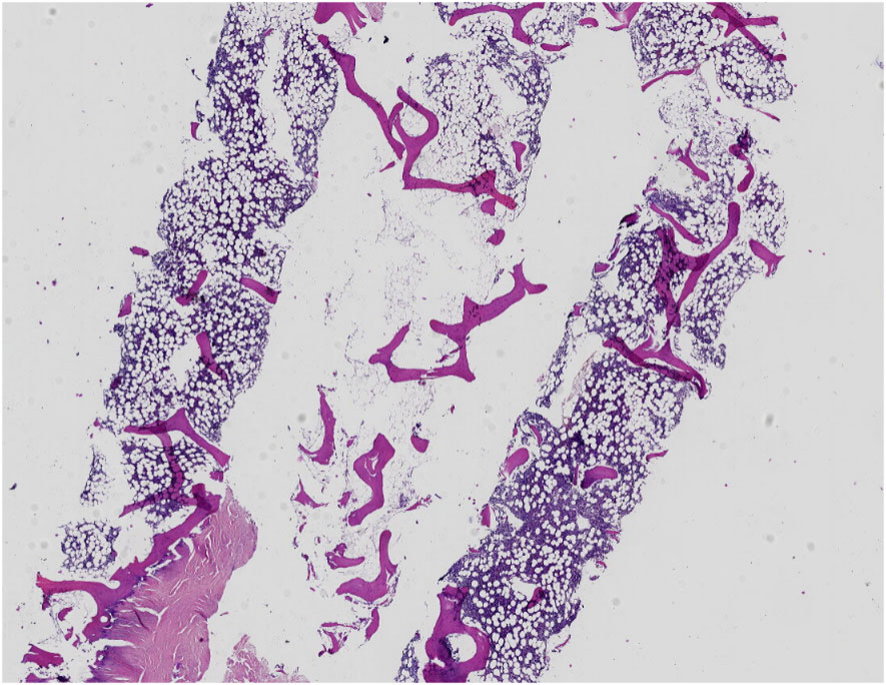
A bone marrow biopsy was negative for malignancy and did not show evidence of hemophagocytosis.

A 36-year-old Chinese man was diagnosed with ankylosing spondylitis due to low back pain and initiated sulfasalazine therapy one month ago. Two weeks after starting sulfasalazine, the patient developed high fever (up to 40°C), facial swelling and generalized erythema, and subsequent cervical lymphadenopathy. Despite receiving antibiotic treatment (ceftriaxone, amoxicillin, and levofloxacin) at a local hospital, there was no improvement in his condition. Laboratory examination revealed leukocytosis (228.9×10^9^/L) and abnormal liver function tests characterized by elevated aspartate aminotransferase (324 U/L; Normal value <50 U/L) and alanine aminotransferase (114 U/L; reference range < 40). Additionally, elevated B-type natriuretic peptide (BNP) levels (1411 pg/ml; normal range: 0-88) were detected along with positive COVID-19 PCR results. The patient was diagnosed with drug hypersensitivity syndrome, drug-induced liver injury, and COVID-19 infection. Subsequently, administration of methylprednisolone (160 mg/day), intravenous immunoglobulin (32.5g/day), and Paxlovid for five days led to relief of high fever and significant subsidence of the rash. Following this initial treatment phase, the patient was transferred to our department for further management. Physical examination showed diffuse exanthematous eruption on the trunk (**Figure 1**). The methylprednisolone dosage was gradually reduced every 3–5 days, during which time most of the skin lesions resolved and the levels of leukocytosis, aspartate aminotransferase, alanine aminotransferase and BNP approached normal ranges. However, after 5 days, the patient experienced an abrupt onset of high fever (up to 40°C) accompanied by abdominal pain. Laboratory evaluation revealed multiple abnormalities, including elevated serum ferritin levels of 11895.00 ng/ml (reference range: 24-336), decreased fibrinogen levels of 1.02 g/L (reference range: 2-4), increased procalcitonin(PCT) levels of 0.61 ng/ml(reference range <0.048), significantly elevated alanine aminotransferase levels of 2462 IU/L (reference range < 50), markedly elevated aspartate aminotransferase levels of 1191 IU/L(reference range < 40), highly increased erythrocyte sedimentation rate (ESR) 109 mm/h (reference range <21), substantially raised CRP level at 145mg/L (reference range <5), extremely high triglycerides concentration at 9.79 mmol/L(reference range: 0.29-1.83). Additionally, the absolute count of NK cells was found to be 32 cells/ul (reference range:154-768). Multiple cytokines were observed to be elevated including interleukin (IL)-2, IL-2R, IL-6, IL-8, IL-10, IFN-gamma(IFN-γ).Tests for antineutrophil cytoplasmic antibody and rheumatoid factor yielded negative results, as did serological tests for hepatitis viruses and human immunodeficiency virus(HIV). EBV DNA was detected at a concentration of 4.11E+05 copies/mL and human CMV was detected at 3.73E+03 copies/mL. Antinuclear antibodies were positive with a titer of 1:1000 speckled pattern. Extractable nuclear antigen antibodies tests showed positive for anti-U1-snRNP antibodies (23.9 AI) and anti-SS-A antibodies (22.7 AI). Thyroid function tests revealed a higher level of reverse triiodothyronine (rT3) at 1.78 nmol/L (normal range: 0.78-1.38), along with a comprehensive thyroid panel including lower levels of triiodothyronine (T3) at 0.64 nmol/L(reference range: 1.3-3.1), free T3 at 1.96 pmol/L(reference range: 3.6-7.5), thyroxine (T4) at 48.60 nmol/L(normal range: 62-164), free thyroxine (fT4) at 11.20 pmol/L(normal range: 12-22), thyroglobulin at a reduced level of only 0.10 ug/L(reference range: 3.5-77), elevated anti-thyroglobulin antibodies at the concentration of 250.00 IU/ml(reference range,<115), and increased anti-thyroid peroxidase antibodies at 44.50 IU/ml(reference range, <34). The abdominal CT scan revealed heterogeneous liver densities, suggestive of intrahepatic lymphatic congestion and a potential liver cyst, as well as an enlarged gallbladder exhibiting edema and splenomegaly (**Figure 2A**). The kidneys displayed irregular borders, accompanied by thickened bilateral renal fascia and parietal peritoneum, as well as a slightly hazy appearance of the surrounding adipose tissue. Additionally, the scan identified an 11 mm dilated appendix that was non-compressible, surrounded by a hazy fat space and several striated shadows in close proximity (see **Figure 2B**). A small amount of pelvic fluid was present. The PET-CT scans did not reveal any definitive evidence of malignancy (**Figure 2C**). Ultrasonographic evaluation of the axillary lymph nodes showed several nodes without significant increase in internal blood flow signal. Furthermore, bone marrow biopsy findings were negative for malignancy and hemophagocytosis indications were absent (**Figure 3**).

The RegiSCAR score was determined to be 7, supporting the diagnosis of DRESS (**Table 1**).^9^ According to the Naranjo adverse drug reaction probability scale, it was probably that sulfasalazine induced this condition. At the same time, our patient fulfilled seven out of the eight required criteria for HLH (**Table 2**).^10^ Ultimately, the patient was diagnosed with sulfasalazine-induced DRES triggering sHLH. Concurrent diagnoses of undifferentiated connective tissue disease and low T3 syndrome were also made. The patient was promptly transferred to the hematology department and initiated on treatment regimen based on the revised diagnostic guidelines for HLH-2004, which included dexamethasone (15 mg/day for 3 days), followed by etoposide (80 mg/day once), and azathioprine (50 mg twice a day). By the third day of therapy, fever subsided while some biochemical parameters normalized. Systemic steroid tapering occurred gradually without any relapse during the two-month follow-up period.

**Table 1:** RegiSCAR scoring system in DRESS for the patient (9).

Criteria	Score				patient score
	−1	0	1	2	
Fever ≥38.5°C	No/unknown	Yes			0
Enlarged lymph nodes		No/unknown	Yes		1
Eosinophilia(× 10^9^/L)		No/unknown	≥ 0.7 × 10^9^/L or ≥ 10% if WBC < 4.0 × 10^9^/L	≥ 1.5 × 10^9^/L or ≥ 20% if WBC < 4.0 × 10^9^/L	
Atypical lymphocytosis		No/unknown	Yes		1
Skin rash extent (% body surface area)		No/unknown	>50% Yes		1
Skin rash suggesting DRESS^a^		No/unknown	Yes		1
Skin biopsy suggesting DRESS	No	Yes/unknown			
Organ involvement Liver Kidney Lung Muscle/heart Pancreas Other organ		No	1 organ	≥ 2 organs	2
Rash resolution ≥ 15 d	No/unknown	Yes			
Excluding other causes^b^		No/unknown	Yes		1
Total score(maximum =9)					7

DIHS, drug-induced hypersensitivity syndrome; DRESS, drug reaction with eosinophilia and systemic symptoms; WBC, white blood cell. Diagnosis made based on total score: < 2 points: not DIHS; 2–3 points: possible DIHS; 4–5 points: probable DIHS; > 5 points: definitive case. Our patient received a score of 7, corresponding to a definite DIHS case.^a^ Suggests DRESS if ≥ 2 purpuric lesions, infiltration, facial edema, psoriasiform desquamation.^b^If ≥ 3 negative: antinuclear antibody, blood culture, hepatitis A virus, hepatitis B virus, hepatitis C virus, chlamydia, mycoplasma.

**Table 2:** Hemophagocytic lymphohistiocytosis diagnostic criteria (10).

HLH-2004 criteria	patient
Fever	yes
Splenomegaly	yes
Cytopenias (affecting 2 of 3 lineages in the peripheral blood): hemoglobin, <9 g/dL; platelets, <100 109/L; neutrophils, <1 109/L	yes
Hypofibrinogenemia (<150 mg/dL) and/or hypertriglyceridemia (fasting >265 mg/dL)	yes
Hemophagocytosis in bone marrow, spleen, lymph nodes, or liver	
Low or absent NK-cell activity	yes
Ferritin >500 ng/mL	yes
Elevated soluble CD25 (soluble IL-2 receptor-a)	yes

Diagnosis of hemophagocytic lymphohistiocytosis is made when at least 5 of the 8 criteria are fulfilled.

## Discussion

In this report, we present a comprehensive case of DRESS syndrome induced by sulfasalazine, which was further complicated by COVID-19 coinfection and concurrent reactivation of EBV and CMV, resulting in sHLH. The diagnosis of DRESS was confirmed based on clinical manifestations including fever, rash, cervical lymphadenopathy, atypical lymphocytes, and involvement of internal organs, such as liver and spleen. The patient’s RegiSCAR score, an important diagnostic tool for DRESS, was calculated to be 7 indicating a high probability of this syndrome (9). We employed the HLH-2004 criteria to diagnose and score HLH (10) in our patient who ffulfilled 7 out of the required 8 criteria. Secondary HLH is a critical condition that can be triggered by various factors. In this case, potential contributors include drug reaction, viral reactivations (EBV and CMV), as well as the patient’s rheumatologic condition and COVID-19 infection. While virus reactivations are observed in over 70% of DRESS cases, not all progress to HLH. Therefore, some researchers have postulated that viruses may not be the primary factor inducing HLH but could contribute to the final prognosis (11). However, limited testing for human herpesvirus 6 (HHV-6) reactivation at our facility hindered a comprehensive understanding of its role in the etiology of this case. Furthermore, the absence of malignancy or evidence suggesting other common triggers complicates our understanding regarding the etiology behind HLH in this particular patient. Since the onset of the COVID-19 pandemic, HLH has been increasingly identified among patients during both acute phase or recovery phase from COVID-19 infection. Similarly, both HLH and Covid-19 are hyperinflammatory syndromes characterized by hypercytokinemia, and share similar pathophysiological mechanisms (12). Therefore, COVID-19 infection played an important role in contributing to the complex condition observed in this patient.

Although the development of DRESS syndrome has been associated with over 50 drugs, reports implicating sulfasalazine as a causative agent are rare, with only a few documented instances (13, 14). It is noteworthy that patients with sulfonamide-induced DRESS often exhibit deficient N-acetyltransferase activity, which may contribute to hypersensitivity reactions (15). Sulfasalazine-induced DRESS complicated by viral activation and subsequent HLH has been infrequently reported in the literature. The first case was documented by Komatsuda et al. in 2008, followed by reports from Liang et al., involving two female patients (8, 16). These cases primarily involved HLH triggered by EBV reactivation. To our knowledge, this is the first reported case of sulfasalazine-induced DRESS coinfected with COVID-19 leading to HLH (17). This highlights the importance for clinicians to be aware of this rare but potentially fatal complication. The complex and multifaceted nature of DRESS syndrome is underscored by its intricate interplay between drug hypersensitivity, viral reactivation, and immune dysregulation.

While the complication of HLH in DRESS syndrome is not uncommon, its pathogenesis is not well understood. Although numerous cases of DRESS and HLH have been reported, most have associated HLH with concurrent infections, providing limited information on the immunologic features or establishing an unclear link between the two diseases. An analysis of 23 cases from the PubMed/MEDLINE database suggested that untreated or persistent DRESS may lead onset of HLH (18). Furthermore, it hypothesized that DRESS and HLH may share a same spectrum of immune dysfunction based on their overlapping characteristics (19). Despite similarities such as activated lymphocytes and hypercytokinemia, the marked and predominant CD8+ T-cell activation observed in HLH is not consistently seen in DRESS (20, 21), which could explain why only certain patients with DRESS develop HLH. Additionally, there may be a lack of recognition of HLH within dermatology practice leading to potential missed diagnoses and an underestimation of the coexistence between these conditions. Timely diagnosis and management are crucial for improving outcomes in patients with both DRESS and associated HLH. Moreover, the exact nature of the relationship between DRESS and HLH remains unclear; further exploration through dedicated disease cohort establishment is required to determine whether DRESS causes or manifests of a shared pathogenic process.

Both DRESS and HLH can result in significant visceral damage, with the liver being the most commonly affected internal organ. However, these diseases can also affect other internal organs such as the heart, lungs, kidneys, and other gastrointestinal tract. Although cholecystitis has been rarely reported in both DRESS and HLH cases (22), appendicitis has not been documented in existing literature. COVID-19 may present gastrointestinal symptoms and has been observed to involve the gallbladder and appendix in a limited number of cases. Acute acalculous cholecystitis and appendicitis are infrequent but clinically significant gastroenterological associations of COVID-19 that can occur without accompanying pulmonary symptoms (23, 24). Our patient exhibited common hepatorenal and splenic involvement, along with rare occurrences of cholecystitis and appendicitis. We hypothesize that excessive immune system activation and cytokine storm resulting from combined DRESS syndrome, COVID-19 infection, and HLH contributed to more extensive systemic damage. This highlights the potential for more serious consequences when DRESS coexists with HLH during a COVID-19 infection.

Endocrine abnormalities in DRESS syndrome typically manifest as delayed sequelae rather than acute symptoms. Thyroid disorders can present in various forms such as thyroiditis or sick euthyroid syndrome (25). A retrospective analysis of 27 patients revealed that five developed anomalies in thyroid function, including sick euthyroid syndrome, thyroiditis, increased free T4 levels and decreased thyrotropin levels (26). Long-term thyroid complications following recovery from DRESS can range from hyperthyroidism to hypothyroidism; antithyroid antibodies often emerge several months after resolution of the condition (27). The presentation of abnormal thyroid function in our patient further underscores the importance of vigilant endocrine follow-up in these cases.

Furthermore, it is imperative to recognize that eosinophilia, which is commonly associated with DRESS syndrome, may not be present in around one-third of cases. This absence is particularly evident among individuals concurrently experiencing hemophagocytic disorders accompanied by leukopenia (28). Our patient’s case exemplifies this observation and emphasizes the need for a thorough and sophisticated diagnostic approach due to the heterogeneous nature of DRESS syndrome presentations. These findings highlight the necessity for clinicians to remain aware of the varied manifestations associated with DRESS syndrome while emphasizing continuous monitoring for potential thyroid dysfunction as part of long-term care.

DRESS is a potentially life-threatening dermatological condition, and the presence of sHLH exacerbates its severity. Early intervention plays a pivotal role in achieving favorable patient outcomes and reducing mortality risk. Pathogenesis, clinical manifestations, and laboratory markers exhibit overlapping features between DRESS and HLH, leading to similarities in their treatment approaches. Whenever feasible, addressing the underlying trigger through appropriate agents targeting infection, malignancy or autoimmune disease should be prioritized as the initial management strategy for sHLH. High-dose corticosteroids are considered first-line therapy for both conditions. Additionally, immunoglobulins and immunosuppressive agents such as methotrexate, azathioprine, and cyclosporine can be used in combination. In severe cases of HLH, etoposide may be employed as a therapeutic option. Furthermore, interleukin-1 inhibitors like anakinra, rilonacept or canakinumab can also be utilized for treating HLH (29). Tocilizumab and JAK inhibitors such as Baricitinib have shown promise in managing macrophage activation syndrome (MAS) associated with COVID-19 infection (30). In this case, initial treatment with high-dose steroids and immunoglobulins failed to yield satisfactory outcomes; however, a significant improvement was observed upon the addition of etoposide and azathioprine.

This case highlights the crucial need for a comprehensive and vigilant differential diagnosis when patients present symptoms suggestive of severe drug hypersensitivity. The correlation between DRESS syndrome and hemophagocytic lymphohistiocytosis (HLH) necessitates heightened awareness and early recognition among clinicians to facilitate timely intervention. In the management of DRESS syndrome, it is advisable to regularly monitor eosinophil levels and conduct serial complete blood counts (CBC), particularly in patients with underlying autoimmune diseases or concurrent COVID-19 infections. These specific conditions can exacerbate the severity or modify the progression of DRESS syndrome and its complications; hence close monitoring and prompt treatment are imperative.

## Data availability statement

The original contributions presented in the study are included in the article/supplementary material. Further inquiries can be directed to the corresponding author.

## Ethics statement

Written informed consent was obtained from the individual(s) for the publication of any potentially identifiable images or data included in this article.

## Author contributions

ML: Data curation, Writing – original draft. FL: Data curation, Writing – original draft. YD: Data curation, Writing – original draft. YZ: Data curation, Writing – original draft. XC: Writing – review & editing.
